# Drunken lipid membranes, not drunken SNARE proteins, promote fusion in a model of neurotransmitter release

**DOI:** 10.3389/fnmol.2022.1022756

**Published:** 2022-10-14

**Authors:** Robert E. Coffman, Katelyn N. Kraichely, Alex J. B. Kreutzberger, Volker Kiessling, Lukas K. Tamm, Dixon J. Woodbury

**Affiliations:** ^1^Neuroscience Center, Brigham Young University, Provo, UT, United States; ^2^Department of Molecular Physiology and Biological Physics, University of Virginia Health System, Charlottesville, VA, United States; ^3^Department of Cell Biology and Physiology, Brigham Young University, Provo, UT, United States

**Keywords:** SNARE proteins, membrane fusion, ethanol, methanol, TIRF, electrophysiology, circular dichroism, FLIC

## Abstract

Alcohol affects many neuronal proteins that are upstream or down-stream of synaptic vesicle fusion and neurotransmitter release. Less well studied is alcohol’s effect on the fusion machinery including SNARE proteins and lipid membranes. Using a SNARE-driven fusion assay we show that fusion probability is significantly increased at 0.4% v/v (68 mM) ethanol; but not with methanol up to 10%. Ethanol appears to act directly on membrane lipids since experiments focused on protein properties [circular dichroism spectrometry, site-directed fluorescence interference contrast (sdFLIC) microscopy, and vesicle docking results] showed no significant changes up to 5% ethanol, but a protein-free fusion assay also showed increased lipid membrane fusion rates with 0.4% ethanol. These data show that the effects of high physiological doses of ethanol on SNARE-driven fusion are mediated through ethanol’s interaction with the lipid bilayer of membranes and not SNARE proteins, and that methanol affects lipid membranes and SNARE proteins only at high doses.

## Graphical Abstract

Low doses of ethanol excite fusion of vesicles to a membrane in a model system of neurotransmitter release. At these same doses, methanol does not increase fusion. Our data demonstrates that ethanol’s effect is mediated through the lipids in the vesicle or planar membrane or both, but not through SNARE proteins (red, cyan, and green lines). 
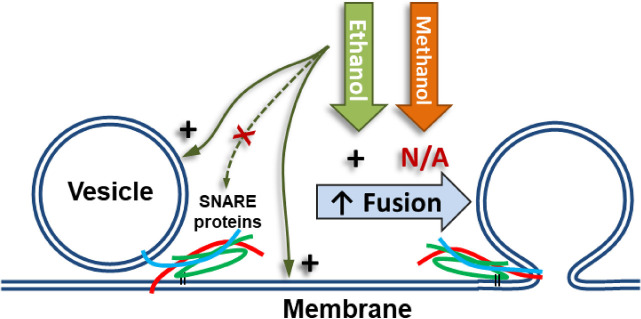

## Introduction

Ethanol use by humans goes back centuries and its consumption produces many effects including relaxation, sleepiness, and intoxication (Vallee, 1994, 1998). Initial symptoms appear above 6 mM blood alcohol content and doses above 86 mM can be fatal (Paton, 2005). After decades of research, the diverse mechanisms of ethanol are still not fully understood. Acute effects of ethanol are thought to originate in the brain, specifically by altering cellular communication, including synaptic transmission (Abrahao et al., 2017).

Cellular communication consists of pre-synaptic events, from action potential to neurotransmitter release, and post-synaptic events, namely, detection of neurotransmitter (NT) and subsequent post-synaptic potential changes. Alcohol has been shown to affect pre-synaptic and post-synaptic proteins (Siggins et al., 2005; Roberto et al., 2006; Barclay et al., 2010). Examples of pre-and post-synaptic proteins known to be affected by alcohol include the large-conductance Ca^2+^-activated K^+^ channel (BK channel) (Abrahao et al., 2017) and many post-synaptic ligand gated ion channels (Lovinger and Roberto, 2013; Howard et al., 2014; Rao et al., 2015; Olsen and Liang, 2017; Söderpalm et al., 2017). Bridging the pre-and post-synaptic events is exocytosis and NT release. Exocytosis requires fusion of membranes that is driven by SNARE (soluble NSF attachment protein receptor) proteins.

In cellular systems, studies have shown that alcohol alters NT release from neurons and neuroendocrine cells. Zhu and Lovinger (2006) showed that in isolated synaptic boutons of the amygdala ethanol enhances release of NT. A thorough review by Das (2020) delineates ethanol’s effect on proteins involved in neurotransmitter release, including; development of tolerance, sensitivity, and regulation of gene and protein expression. Shaaban et al. (2019) showed that intracellular methanol rescues endocrine release from chromaffin cells that have deficient fusion due to a mutation in a SNARE protein. Such cellular studies implicate alcohol’s direct effect on exocytosis and neurotransmitter release, but do not confirm it.

This report focuses on exocytosis; pre-and post-synaptic events in cells are not considered further [for several reviews on the topic see Abrahao et al. (2017), Cui and Koob (2017), Harrison et al. (2017), Alasmari et al. (2018), Lovinger (2018), Das (2020), and Roberto et al. (2021)]. Exocytosis requires the folding and assembly of the SNARE complex and ultimately fusion of vesicle and cell membranes. These SNARE proteins are the minimal machinery needed to provide the energy for membranes to fuse (Weber et al., 1998; Gao et al., 2012; Kiessling et al., 2018; Tian et al., 2019). The neuronal SNARE proteins syntaxin-1a (syx) and SNAP-25A (SNAP-25) are associated with the plasma membrane while synaptobrevin-2 (syb2) is associated with the vesicle membrane (Söllner et al., 1993; Brunger et al., 2019). Reconstituting these SNARE proteins into simple model membranes allows the direct observation of ethanol’s effect on the exocytotic event without the complications of other proteins or charged lipids present in cellular systems. A single model synapse system as discussed in Woodbury (1999) that has both a pre and post-synaptic membrane with their respective minimal components is yet to be developed, but models of the pre-synaptic membrane have been discussed and demonstrated (Tamm et al., 2003; Domanska et al., 2009; Kiessling et al., 2017) and are expected to have pharmacologically relevant properties to neurons. We employed several model systems to understand the effects of alcohols directly on membrane fusion, that last step of exocytosis with its accompanying proteins, without the influence of other pre-and post-synaptic events that occur in cell based assays.

In model systems formed of lipid bilayers only (i.e., protein-free) researchers have found that alcohols affect membrane properties in multiple ways. Specifically, increasing alcohol concentrations can do three things: (1) increase vesicle fusion rates (Paxman et al., 2017), (2) reduce the membrane tension required to lyse vesicles (Ly and Longo, 2004; Wittenberg et al., 2008), and (3) increase the probability of a hemifusion state, where only the closer leaflets of a vesicle and a planar lipid bilayer mix (Chanturiya et al., 1999). Many studies have also shown that lipid composition can alter the process of membrane fusion and lipid bilayer properties (Chernomordik et al., 1985; Lang et al., 2001; Churchward et al., 2005, 2008; Tong et al., 2009; Domanska et al., 2010; Ge et al., 2010; Wang et al., 2010; Koseoglu et al., 2011; Lee et al., 2013; Kreutzberger et al., 2015, 2017b; Stratton et al., 2016; Yang et al., 2016; Kiessling et al., 2018). Since ethanol and methanol interact with lipid bilayers, and because lipid composition (particularly charged lipids and cholesterol) can alter the process of membrane fusion, alcohols may alter fusion by effectively modifying relevant membrane properties.

In this report we focus on ethanol’s acute effect in a reduced reconstituted system and its individual components, that mimic neurotransmitter release (exocytosis) and asked the question: Does alcohol change exocytosis by affecting the SNARE proteins or the lipid bilayer of fusing membranes (or both)?

## Materials and methods

### Purification of proteins

For use in CD spectrometry experiments, full length (1–116) synaptobrevin-2 (syb2), and syntaxin-1a containing only the SNARE and the membrane spanning domains (syx, 194–288) was produced from *Escherichia Coli* containing a pGEX KG plasmid that codes for a GST-syb2 or GST-syx fusion protein and ampicillin resistance genes (Woodbury and Rognlien, 2000). SNAP-25 protein purification used for CD spectrometry, and cysteine modified SNAP-25 (dSNAP) used for TIRF microscopy is described below. Starter cultures (2 × 5 mL) of 2xYT broth containing 200 μg/mL ampicillin were inoculated with the proper *E. Coli* and grown at 37°C for 12 h (OD_600_∼1.0). Then 2.5 mL of starter culture were added into four flasks containing 500 mL of 2xYT broth and 100 μg/mL ampicillin. All flasks were incubated at 28°C for 5 h. After 5 h, protein expression was induced by addition of Isopropyl-β-D-thiogalactopyranoside (IPTG, 30–150 μM final). The flasks were incubated about four more hours to a final OD_600_ of 2–3, and the cells were pelleted (7,500 RCF at 4°C for 7 min). The supernatants were removed and pellets combined and suspended in 20 mL buffer A (300 mM KCl, 10% w/v glycerol, 50 mM Tris–HCl, pH 8.0 with KOH) supplemented with 5 mL of 20% TX-100 and 3.6 μL of neat β-Mercaptoethanol (βME, 2 mM final concentration). After gentle mixing, the bacteria were lysed by running through a French press at 16,000–24,000 psi and 1 Protease inhibitor tablet was added (Pierce Mini Tablets #A32953) to the ∼25 mL lysate. The lysate was clarified by centrifugation (13,500 RCF at 4°C for 45 min) and the supernatant was added to 1 mL of 50% glutathione bead slurry (Sigma Chemical Co., St. Louis, MO, United States) and incubated on a rotary shaker at RT for 2 h. The beads were pelleted (60 RCF at RT for 45 s), and the supernatant removed and discarded. The beads were washed 4 more times with 10–20 volumes of buffer A containing 0.2% TX-100 and 2.5 mM βME and then washed three more times with buffer B (100 mM KCl, 8 mM CHAPS, 50 mM Tris–HCl, 10% v/v glycerol, pH 8.0 with KOH) and 10 mM βME. After the last wash, 1.5 mL Buffer B with βME were added to the suspended beads and the protein was clipped from the GST portion by adding 10 μL of Thrombin (1.0 U/μL) (Sigma Chemical Co., St. Louis, MO, United States) and incubating on a rotary shaker for 2.5 h at RT. AEBSF (2.5 mM final) was added to inactivate thrombin, and the tube was left on the rotary shaker for 30 more min. The tube containing glutathione beads and protein was then centrifuged according to bead manufacturer’s recommendations and the protein-containing supernatant was removed and stored as a 600 μL aliquot. The glutathione beads were washed a second time in the same buffer, resulting in two 600 μL aliquots of purified protein per culture grown.

Ammonium sulfate precipitation was used to further purify syb2. Multiple batches of protein were combined and solid ammonium sulfate was added until the solution reached 20% (w/v), it was rocked at 4°C for 1 h and the solution centrifuged (10,000 RCF at 4°C for 15 min) to remove thrombin and GST. Ammonium sulfate was added to 30% and the process was repeated to precipitate syb2. The precipitated syb2 was re-suspended in Buffer C (20 mM potassium phosphate buffer, pH 7.2) and ammonium sulfate was removed by dialysis against Buffer C. Aliquots of the protein were stored at −80°C until used.

Acid precipitation was used to further purify syx. Multiple batches of protein were combined and the protein titrated to pH 4.7 with 1 M Acetic Acid. Solution was spun for 30 min at 4°C at 10,000 RCF to pellet the protein. Supernatant was discarded and the pellet was resuspended in Buffer C. Aliquots of the protein were stored at −80°C until used.

SNARE proteins for the sdFLIC experiments and SNARE fusion assays were expressed and purified as previously reported (Liang et al., 2013; Kreutzberger et al., 2016). Syx (residues 183–288), wild-type SNAP-25A, and syb2 from *Rattus norvegicus* cloned into a pET28a expression vector under the control of a T7 promoter were expressed in *Escherichia coli* strain BL21(DE3) cells. Bacteria were grown at 37^°^C until an optical density of 0.6–1.0, then induced with 1 mM IPTG and grown overnight at 20^°^C.

For syx, bacteria were then pelleted by centrifugation and resuspended in buffer H500 (20 mM HEPES, 500 mM NaCl, pH 7.4) containing protease inhibitor cocktail before being lysed by sonication. Membrane fractions were collected from the lysate using ultra centrifugation and protein was extracted from the pellet with 2% Triton-X and 6 M urea. After several hours of incubation, membrane debris was removed by ultracentrifugation and the supernatant was applied to nickel-nitrilotriacetic acid (Ni-NTA) affinity chromatography column. The column was washed extensively to remove urea, and detergent was exchanged to 0.2% Dodecylphosphocholine (DPC) before elution with imidazole. After removal of the His_6_ tag using bovine thrombin, syx was further purified by applying it to a Superdex200 size exclusion column and collecting the fraction for monomeric syx in a DPC micelle.

For SNAP-25, bacteria were then collected by centrifugation and resuspended in buffer H500 containing protease inhibitor cocktail before being lysed by sonication. Membrane fractions were removed by ultracentrifugation and the supernatant was directly applied to a Ni-NTA column. After repeated washing steps, the protein was eluted with imidazole. Eluted fractions were pooled and combined with thrombin to remove the His_6_-tag while being exchanged into buffer with 50 mM NaCl *via* overnight dialysis. Following this, SNAP-25 was further purified using a MonoQ ion exchange column. For CD spectrometry experiments, SNAP-25 was dialyzed overnight in Buffer C. For TIRF microscopy experiments, SNAP-25 was quadruply dodecylated through disulfide bonding of dodecyl methanethiosulfonate to its four native cysteines to mimic the native lipid anchoring of SNAP-25 in mammalian cells (Kreutzberger et al., 2016).

For syb2, cells were collected and resuspended in detergent-free buffer HID (20 mM HEPES, 500 mM NaCl, 20 mM imidazole, 5 mM DTT, pH 7.4) with protease inhibitor cocktail. One volume of the same buffer but containing 25% sodium cholate was added for every four volumes of the resuspended cell pellet. Cells were then lysed by sonication followed by the addition of 6 M urea. Membrane debris was pelleted and supernatant was added to a Ni-NTA column. After several washes, detergent was exchanged to 0.1% DPC and protein was eluted with imidazole. Following cleavage of the His_6_ tag with thrombin, protein was further purified using a Superdex200 size exclusion column. For all protein samples, purity was verified using SDS-PAGE.

### Reconstitution of synaptobrevin-2 into proteoliposomes

Proteoliposomes containing syb2 at a lipid to protein ratio of 400:1 were made as previously described (Domanska et al., 2009; Kreutzberger et al., 2016) with lipid compositions of 79:20:1 POPC:cho:Cy5-DOPE. Lipids were mixed and organic solvents were evaporated under a stream of N_2_ gas followed by vacuum for at least 1 h. The dried lipid films were dissolved with 25 mM sodium cholate in buffer H150 (20 mM HEPES, 150 mM KCl, pH 7.4) followed by the addition of an appropriate volume of syb2 in 0.1% DPC to reach a final volume of 180 μL. After 1 h equilibration at room temperature, the mixture was diluted below the critical micellar concentration by adding more buffers to a final volume of 550 μL and sample was dialyzed overnight against 500 mL buffer H150 at 4^°^C with one buffer change.

### Reconstitution of syntaxin-1a and SNAP-25 into planar supported bilayers

Proteoliposomes containing syx and dodecylated SNAP-25A (dSNAP) at a lipid to protein ratio of 3,000 were made exactly as described for syb2 but with a lipid composition of 80:20 POPC:Cholesterol. These syx:dSNAP proteoliposomes were then used to form planar supported bilayers using the Langmuir-Blodgett/vesicle fusion technique (Kalb et al., 1992; Wagner and Tamm, 2000). Quartz slides were cleaned in 1:3 hydrogen peroxide (30%):sulfuric acid by volume for 20 min followed by extensive rinsing with milliQ water. The first leaflet of the bilayer was then formed by Langumir-Blodgett transfer directly onto the quartz slide using a Nima 611 Langmuir-Blodgett trough (Nima, Conventry, UK) by applying the lipid mixture of 80:20 POPC:cholesterol from a chloroform solution. After allowing the solvent to evaporate for 10 min, the monolayer was compressed at a rate of 10 cm^2^/min to reach a surface pressure of 32 mN/m. After equilibration for 10 min, a clean quartz slide was rapidly (68 mm/min) dipped into the trough and slowly (5 mm/min) withdrawn, while a computer maintained a constant surface pressure and monitored the transfer of lipids oriented toward the hydrophilic substrate. Then 300 μL of syx:dSNAP proteoliposomes (100 μM total lipid) were incubated over the bilayer for 1 h in buffer H150 at room temperature to form the second leaflet of the supported bilayer with >80% of the protein complexes oriented so that the SNARE domains point away from the glass substrate (Kiessling et al., 2017).

### Circular dichroism spectrometry of SNARE proteins

For each experiment, three equimolar samples of protein were prepared in 1.5 mL polypropylene micro tubes (Sarstedt, Inc., Newton, NC, United States); 0, 2, and 10% alcohol, (sometimes with 0, 1 and 5% alcohol) with 20 mM phosphate buffer pH 7.1, and 150 mM NaF. Before the first scan, cuvettes were acid washed for 5–10 min using 3N HCl in 50% (v/v) ethanol or washed with cuvette cleaner (Starna Cells, Inc., Atascadero, CA, United States) and rinsed thoroughly with dH_2_O and then dried using an ethanol rinse with subsequent N_2_ gas flow for 30–60 s (gas flow was continued 10–15 s after ethanol was no longer visible in the cuvette). A scan was taken of the three prepared samples in different cuvettes (after a baseline scan of water for each cuvette was obtained). The subsequent 0.4, 1, and 5% (or 0.4 and 2%) alcohol samples were prepared by mixing the 0 with 2% (or 0 with 1%), the 0.4 with 2% (or 1 with 5%), and 0.4 with 10% alcohol samples, respectively. Each newly mixed sample was scanned in a cuvette that already had a lower dose sample removed, without rinsing or washing. A Circular Dichroism Spectrophotometer (Aviv model 420) was used with a 0.1 cm quartz cuvette filled with 280–320 μL of sample at 21°C. Spectra were obtained scanning every nm from 260 to 185 nm and averaging 5–30 s/nm. Dynode voltage was monitored and protein concentrations were used so that the voltage did not go above 600 V.

### Circular dichroism spectrometry analysis

For all samples, a baseline spectrum was subtracted and spectra from 250 to 200 nm were analyzed using the webserver BeStSel Multiple Spectra Analysis (Micsonai et al., 2015) which deconvolutes the CD spectra into 8 structural categories. Our “Helix” category is the sum of Helix1 and Helix2. The “Beta” category is the sum of Anti1, Anti2, Anti3, and Para. The “Other/Turn” category is the sum of Turn and Others. Data are plotted as changes in secondary structure compared to the structural composition of the 0% alcohol sample.

### Protein-free fusion assay

Fusion of liposomes to a planar lipid bilayer (BLM) were measured exactly as described previously (Paxman et al., 2017). Presented data are a combination of previously published data and new data. Briefly, liposomes containing 4:1:1:2 mol ratio of phosphatidylethanolamine (PE), phosphatidylcholine (PC), phosphatidylserine, and ergosterol were formed in the presence of 50 μM nystatin. BLM were formed from a 20 mg/ml decane solution containing a 7:3:3 mol ratio of PE, PC, and cholesterol. Fusion of liposomes to the BLM was induced by adding extra KCl on the vesicles (*cis*) side of the membrane to form a transmembrane osmotic gradient of ∼0.46 OsM. Fusion of individual liposomes to the BLM caused a brief increase in membrane conductance due to nystatin channels and was detected using standard electrophysiology equipment. Fusion rates after addition of alcohol were normalized to fusion rates in the same experiment before addition of alcohol.

### Total internal reflection fluorescence microscopy

Experiments were carried out on a fluorescence microscope (AxioObserver Z1, Carl Zeiss, Thornwood, NY, United States), equipped with a 63x water immersion objective (Zeiss; N.A. = 0.95) and prism-based TIRF illumination. The light source was an OBIS 640LX laser from Coherent Inc., (Santa Clara, CA, United States). Fluorescence was observed through a 665 nm long pass filter (LP665; Semrock, Rochester, NY, United States) by an electron multiplying CCD (DV887ESC-BV; Andor Technologies, Belfast, United Kingdom). The EMCCD was cooled to −70^°^C, and the gain was typically set to an electron gain factor of 200. The prism-quartz interface was lubricated with glycerol to allow easy translocation of the sample cell on the microscope stage. The beam was totally internally reflected at an angle of 72 from the surface normal, resulting in an evanescent wave that decays exponentially with a characteristic penetration depth of ∼100 nm. Which means that vesicle fluorescence is not visible until they approach the membrane. An elliptical area of 250 x 65 μm was illuminated. The laser intensity, shutter, and camera were controlled by a homemade program written in LabVIEW (National Instruments, Austin, TX, United States).

### Single vesicle fusion assay

Synaptobrevin-2 (Syb2) proteoliposomes labeled with 1 mol% Cy5-DOPE lipid were injected, at a concentration of ∼100 nM lipid, onto the syx:dSNAP containing planar supported bilayers. The fluorescence of the proteoliposomes was recorded by TIRF microscopy using a 640 nm laser after focusing the microscope in the first 30 s after injection of syb2 proteoliposomes. Movies were acquired for 3,000 frames collected every 20 ms from 4 to 5 experiments per condition.

Single-vesicle fusion data were analyzed using a homemade program written in LabVIEW (National Instruments). Stacks of images were filtered by a moving average filter. The intensity maximum for each pixel over the whole stack was projected on a single image. Vesicles were located in this image by a single-particle detection algorithm described in Kiessling et al. (2006). The peak (central pixel) and mean fluorescence intensities of a 5 × 5 pixel^2^ area around each identified center of mass were plotted as a function of time for all particles in the 10,000 images of each series. The exact time points of docking and fusion were determined from the time of docking to the time of fusion for individual fusion events and the fusion efficiency was determined from the number of vesicles that fused compared with the total number of vesicles that docked for each bilayer. Since we previously reported that 65% of docked vesicles fuse within 250 ms, vesicles that did not fuse within 1s of docking were considered as part of the non-fused population. Complexes and single SNAREs that exhibited very low docking activity had very few to no events observed at single liposome concentrations and were not analyzed in detail for fusion, except to verify that there was no fusion occurring in the single SNARE cases.

### Total internal reflection fluorescence microscopy binding assay

Docking of syb2 proteoliposomes was performed by injecting 5 μM lipid in 1 mL of buffer into the planar supported bilayer chamber. Images were taken every 30 s to determine the amount of fluorescence in the TIRF field. The first few images were taken immediately before injection to establish the baseline. The mean intensity per pixel was recorded and used to compare binding amounts under different concentrations of alcohols. The intensity over time curves was fit with an exponential first order kinetic curve to determine the saturation intensity for each experiment. Because the fluorescence intensity changes when the fluorophores are transferred from the spherical liposomes to the planar supported membrane due to dequenching and changes of the fluorophores’ orientation relative to the polarized evanescent wave (Kiessling et al., 2010), the determined intensities are corrected for each condition. The observed intensity *I*_*obs*_ is the sum of fluorescence originating from unfused liposomes and from liposomes that underwent fusion:


$$I_{o\,b\,s}=N\,\left(\left(1-\alpha \right)\,i_{l\,i\,p\,o\,s\,o\,m\,e}+\alpha \,\beta \,i_{l\,i\,p\,o\,s\,o\,m\,e}\right)$$

with the total number of bound liposomes *N*, the fusion efficiency α, the intensity from a single liposome before fusion i_liposome_, and the intensity increase due to fusion β. With the normalization constant *K* overall binding of liposomes is reported according to:


$$\hat{N}=K\,\frac{I_{o\,b\,s}}{1+\alpha \,\left(\beta -1\right)}$$

From single fusion events we observed an intensity increase of β = 2, and correction factors 1/(1+α) between 0.74 for the lowest observed fusion efficiency and 0.63 for the highest observed fusion efficiency. Average amount of binding for each condition was determined from 3 experiments.

### Site-directed fluorescence interference contrast microscopy assay

Syntaxin-1a (Syx) with a single cysteine at residue 192 was labeled with alexa546 and assembled with SNAP-25 (all four cysteines mutated to serines) and syb2 (1–96) to form a ternary SNARE complex. This complex mimics the conformation of Syntaxin in a potential prefusion trans-SNARE complex and it has been shown previously that its conformation (characterized by the z-distance of residue 192 to the membrane surface) correlates with fusion activity when altered by Ca2+ mediated membrane binding of C2 domains and/or changes in acyl-chain order (Kiessling et al., 2018). After reconstitution into a supported membrane consisting of 80% POPC and 20% cholesterol, the z-distance changes to the membrane of the labeled residue 192 upon addition of alcohol was measured by site-directed fluorescence interference contrast (sdFLIC) microscopy as previously reported (Kiessling et al., 2018).

The principle of sdFLIC experiments and the set up as used in this work, has been described previously (Liang et al., 2013). A membrane containing protein with specifically labeled cysteines is supported on a patterned silicon chip with microscopic steps of silicon dioxide. The fluorescence intensity depends on the position of the dye with respect to the standing modes of the exciting and emitting light in front of the reflecting silicon surface. The position is determined by the variable-height 16 oxide steps and the constant average distance between dye and silicon oxide (Lambacher and Fromherz, 2002).

Images were acquired on a Zeiss Axioskop fluorescence microscope (Carl Zeiss) with a mercury lamp as a light source and a 40× water immersion objective (Zeiss; N.A. = 0.7). Fluorescence was observed through a 610-nm band-pass filter (D610/60; Chroma) by a CCD camera (Orca-ER, Hamamatsu, Bridgewater, NJ, United States). Exposure times for imaging were set between 100 and 2,000 ms, and the excitation light was filtered by a neutral density filter (ND 1.0, Chroma) to avoid photobleaching.

Starting with buffer H150, the ethanol or methanol concentrations were increased stepwise up to 10%. 10–20 min after each buffer change 4–6 images were acquired for each condition of one supported membrane. From each image, we extracted 100 sets of 16 fluorescence intensities and fitted the optical theory with the fluorophore-membrane distance as fit parameter. Software to fit the data was kindly provided by the authors of Lambacher and Fromherz (2002). The standard deviation of these ∼400–600 results were usually in the order of 1 nm. The optical model consists of five layers of different thickness and refractive indices (bulk silicon, variable silicon oxide, 4 nm water, 4 nm membrane, bulk water), which we kept constant for all conditions (Kiessling and Tamm, 2003; Crane et al., 2005; Liang et al., 2013). Results of the distance changes due to alcohol relative to no alcohol in the buffer are reported for six repeats together with the mean distance changes.

## Results

**FIGURE 1:**
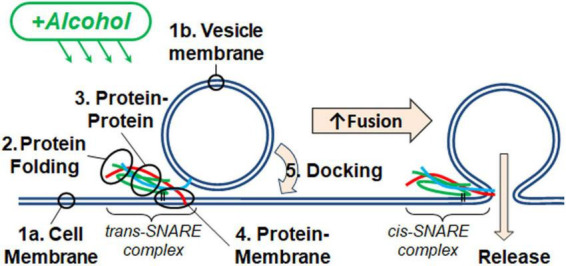
Alcohol could alter fusion by acting at one or more of the five numbered targets. A SNARE-driven fusion assay was used to assess the effects of methanol and ethanol on this reduced fusion system. Other techniques with fewer components targeted one or more of the possible sites of alcohol action including: (1a,b) membranes, (2) SNARE protein folding, (3) protein-protein interactions, (4) protein-membrane interactions detected by the transition from *trans*- to *cis*-(mimicking) SNARE complex, and finally (5) vesicle docking. Membranes are shown as double blue lines; SNARE proteins are syntaxin (red), SNAP-25 (green), and synaptobrevin (cyan).

A SNARE-driven fusion assay was used to measure the effect of methanol and ethanol on fusing membranes in the presence of SNARE proteins (Figure 1). Several measurements of alcohol’s effect on SNARE proteins in relation to exocytosis were used, including: circular dichroism (CD) spectrometry, site-directed fluorescence interference contrast (sdFLIC) microscopy, and TIRF microscopy. In addition, a protein-free fusion assay was used to measure the effects of methanol and ethanol on fusing membranes without proteins. Results with each assay are reported below.

### SNARE-induced fusion

The SNARE-mediated single vesicle fusion assay quantitates the fusion probability between lipid labeled synaptobrevin-2 (syb2) liposomes and planar supported bilayers containing syntaxin-1a (syx) and dodecylated SNAP-25A (dSNAP) (Domanska et al., 2009). This assay detects 100’s of docking events in a single experiment (see Supplementary Movie 1 and Supplementary Figure 1). This assay allowed us to examine if alcohols have any direct effect on SNARE-driven fusion in a reconstituted model system shown to mimic neuronal exocytosis.

**FIGURE 2:**
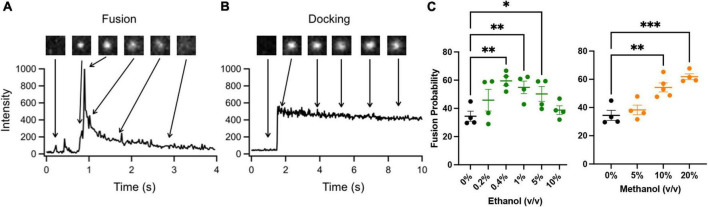
SNARE-driven fusion assay quantifies fusion probability in the presence of alcohol. **(A,B)** the top boxes are fluorescence images of a single vesicle at the corresponding time in the line graph. The line graph represents the peak fluorescence intensity originating from the vesicle site. **(A)** Shows a single docking and fusion event as a large spike in fluorescence intensity with subsequent rapid decay as the labeled lipids diffuse away. **(B)** Shows a docking event as a sudden jump in fluorescence intensity with no subsequent decay indicating that fusion did not occur. **(C)** Fusion probabilities determined in the presence of buffer with different concentrations of ethanol (green) or methanol (orange) compared to controls (black). Notice that 0.4% v/v (68 mM) ethanol significantly increases fusion probability, while 0.2% (34 mM) shows an intermediate fusion probability but is not significantly different from control. Methanol does not show an increase in fusion probability until 10%. Fusion probability is expressed as % of docking events that undergo fusion, (i.e., successful fusion/total docking events). Error bars are 95% confidence intervals, *, ^**^, and ^***^ indicates *p* ≤ 0.05, *p* ≤ 0.01, and *p* ≤ 0.001 respectively compared to controls (one-way ANOVA, *n* ≥ 4).

Figure 2 shows two docking events; one resulting in fusion (A) and the other no fusion (B), and fusion probability results (C) from the SNARE-driven fusion assay. Panel C shows that ethanol and methanol are effective at increasing fusion probability. However, ethanol is >35 times more potent, producing a significant increase at 0.4% v/v compared to methanol at 10%. A dose of 0.4% ethanol is 68 mM (or 0.31% w/v) and below the 86 mM maximum physiologically relevant range. The SNARE-fusion assay also shows that ethanol is less effective at 0.2% (34 mM) than 0.4% but loses its effect at higher doses, as discussed in a later section (Experimental Considerations). This confirms that this simple model of exocytosis is responsive to physiologically relevant doses of alcohol and distinguishes between ethanol and methanol. Since this model system still has several components, we reduced the problem further and tested individual parts to see if we could elucidate which components, (SNAREs or lipids, see Figure 1) were responsible for mediating this effect. We started with the SNARE proteins.

### Effect of alcohol on SNARE proteins and protein/membrane interactions

**FIGURE 3:**
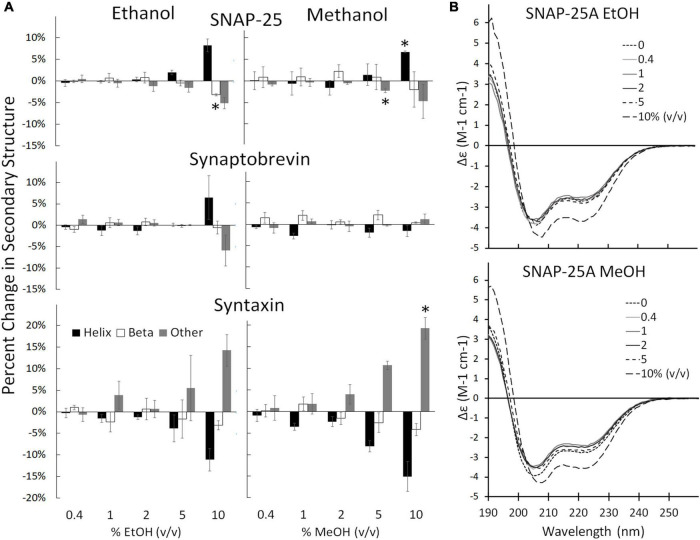
Secondary structure changes of the three SNARE proteins as determined by CD spectrometery. **(A)** Shows the SNARE proteins (from top to bottom: SNAP-25, syb2, and syx) when treated with ethanol (left) and methanol (right). The three SNARE proteins show no change in their secondary structure significantly at the low ethanol doses seen to cause significant functional effects in the SNARE-driven fusion assay. An alcohol-induced change in secondary structure is only significant at 10% for syx and above 5% v/v for SNAP-25. **(B)** Shows representative CD spectra of SNAP-25 with increasing doses of ethanol (top) and methanol (bottom). Similar spectra were obtained with syx and syb2 (Supplementary Figures 2, 3 respectively). Error bars are SEM, ^*^p < 0.05 using two-tailed Student’s *t*-test. *n* = 2 for all 10% doses. *n* = 2–4 for all other doses.

Individual SNARE proteins are thought to be relatively unstructured until their SNARE domains associate to form helixes that wind together to form a coiled coil (Fasshauer et al., 1998; Sørensen et al., 2006; Saikia et al., 2021). Helicity increases progressively through three steps: (1) as SNAP-25 and syntaxin bind to form the acceptor complex on the cell membrane, (2) as the acceptor complex zippers with syb2 from the vesicle to form the *trans* SNARE complex (also known as docking), and (3) as zippering continues through the membrane-spanning domains which increases helicity, induces fusion, and forms the *cis* SNARE complex (Fasshauer et al., 1997; Hanson et al., 1997; Witkowska et al., 2021). Formation of the full *cis* complex provides the energy for fusion. We hypothesized that alcohols could enhance fusion by promoting individual SNARE domains to transition from unstructured to helix thus promoting SNARE complex formation. Using CD spectrometry, we show that the secondary structure of the SNARE proteins SNAP-25, syx, and syb2, are stable in the presence of methanol and ethanol below 5% alcohol (Figure 3, Supplementary Figures 2, 3). This is in sharp contrast to results from the SNARE-driven fusion assay that shows enhanced fusion at 0.4% v/v ethanol [and surprising considering our preliminary results that pH, salt and temperature significantly affect SNAP-25 structure (Sumsion et al., 2022)]. One would expect that if alcohol were increasing fusion probability *via* its effects on protein structure that these relatively unstructured individual proteins would experience secondary structural changes at the doses of alcohol seen to increase fusion probability, which is not the case for ethanol. However, this appears to be true for methanol, which causes an increase in Helix (of SNAP-25) and fusion probability at 10% methanol. This is not consistent with our hypothesis that a physiological dose of ethanol increases fusion probability by increasing helicity of SNARE proteins (or by any change in secondary structure).

**FIGURE 4:**
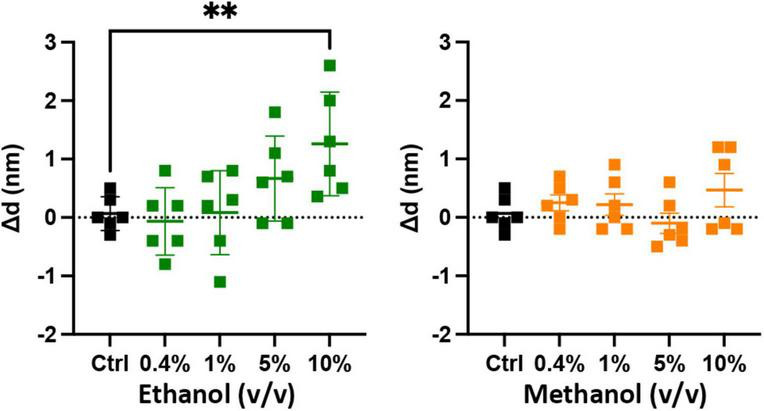
Low doses of alcohols do not cause a measurable change in a membrane anchored SNARE complex. The sdFLIC microscopy experiments detect distance changes (Δd, N-terminus of syx SNARE motif to membrane surface), i.e., an orientational change from a trans-like to a *cis*-like SNARE complex with a more rigid linker between syntaxin’s SNARE motif and trans-membrane domain. When caused by changes in membrane order, this change correlates with enhanced fusion probability (Kiessling et al., 2018). However, no significant changes in SNARE orientation were detected following addition of up to 5% ethanol and up to 10% methanol. Shown are the changes in the distance of syx*192-Alexa546 to the membrane surface compared to control (see Supplementary Figure 4 for example images, data fit, and histograms) [^**^p < 0.01 (one-way ANOVA), *n* = 6].

The results of Figure 3 show that ethanol is not promoting membrane fusion by affecting the secondary structure of individual SNARE proteins; however, ethanol may affect protein-protein interactions that lead to SNARE complex formation or may alter protein-lipid interactions necessary for membrane fusion. We used sdFLIC microscopy to measure the effect of alcohols on SNARE-SNARE and SNARE-lipid interactions. Previously, we have shown that a more rigid linker between the trans-membrane domain and the SNARE domain of syx correlates with the transition from *trans*- to *cis*-SNARE complex (see Figure 1) and with increased fusion probability. Rigidity of the linker was measured as an increase in distance from the membrane to a fluorescence label (Alexa546) attached near the N-terminus of syx’s SNARE domain (residue 192) in complex with SNAP-25 and syb2 (1–96) in different lipid environments (Kiessling et al., 2018). One would expect that if alcohols were promoting fusion through SNARE-SNARE or SNARE-lipid interactions, that the distance of the probe from the membrane would likewise change. As shown in Figure 4, only at 10% ethanol was a significant distance increase seen (see Supplementary Figure 4 for example images, data fit, and histograms). Thus, the SNARE structural data from CD (Figure 3) and sdFLIC experiments (Figure 4) both agree that only unphysiologically high doses of alcohols (≥5%) can alter protein structure in a way that may promote membrane fusion. These results do not agree with the hypothesis that physiological doses of alcohol changes SNARE-SNARE or SNARE-lipid interactions to promote increased fusion probability as seen in Figure 2.

### Effect of alcohol on vesicle docking

**FIGURE 5:**
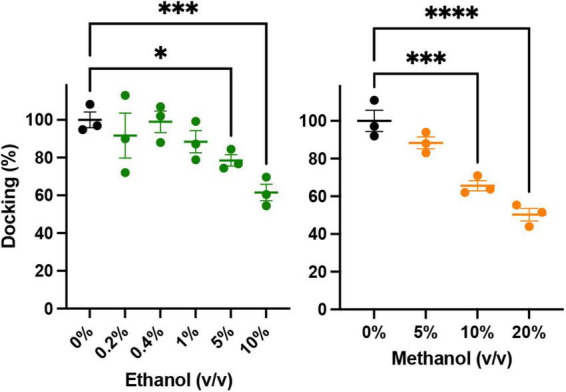
Low concentrations of alcohols do not alter vesicle docking. Using TIRF microscopy, docking of reconstituted vesicles was measured and found to not significantly be affected by methanol or ethanol until ≥5% v/v was added. Error bars are 95% confidence intervals, ^*^, ^***^, and ^****^ indicates *p* ≤ 0.05, *p* ≤ 0.001, and *p* ≤ 0.0001 respectively compared to controls (one-way ANOVA, *n* = 3) Supplementary Figure 5 shows example binding data with the fitted exponential first order kinetic curve.

An important precursor to vesicle-membrane fusion is vesicle docking. Docking proceeds in two steps: loose and then tight docking (Witkowska et al., 2021). Docking is aided by SNARE complex formation. Our SNARE-driven docking assay quantifies 100s of docking events through increasing fluorescence as a result of immobilized fluorescently labeled vesicles within the evanescent field of the TIRF microscope. If docking were changed, it may explain why we see an increase in fusion probability in the SNARE driven fusion assay. For example, if alcohol were to increase clustering of the acceptor complex (syx and dSNAP) in the target membrane, there would effectively be a decrease in the density of docking sites, which would decrease overall docking. In this scenario, there would be more SNARE complexes available at each docking site, which would increase fusion probability similar to what is shown in Figure 2. Such an effect is suggested by evidence that the general anesthetics propofol and etomidate decrease syx mobility in PC12 cells (Bademosi et al., 2018) and may also explain the effect we see with ethanol. Results from our docking experiments are shown in Figure 5. These data make it clear that in the presence of SNARE proteins, docking decreases at unphysiologically high (>5%) alcohol concentrations, but is not changed at physiologically relevant (<86 mM = 0.50% v/v = 0.40% w/v) concentrations, suggesting that ethanol does not affect SNARE-SNARE interactions in the process of docking and therefore does not explain the increased fusion probability at 0.4% ethanol seen in Figure 2.

### Effect of alcohol on lipid bilayers

So far, we have shown that <1% ethanol, but not methanol, increases fusion probability in a SNARE-driven fusion assay (Figure 2) and that these doses of ethanol do not significantly affect SNARE protein secondary structure (Figure 3), SNARE-lipid interaction or SNARE orientation (Figure 4), or docking (Figure 5). These data are summarized in the first four data rows of Table 1 and leads to the conclusion that high physiological doses of ethanol increase fusion through purely lipid interactions. Indeed, this is consistent with our previous results using a protein-free fusion assay.

**TABLE 1:** Summary of effects of ethanol and methanol on components involved in fusion.

Assay	Interactions tested					Result/observation			
	1. Membrane-membrane	2. Protein folding	3. Protein-protein	4. Protein-membrane	5. Vesicle docking	Low ethanol (≤ 1%)	High ethanol (≥ 2%)	Low methanol (≤ 2%)	High methanol (≥ 10%)
SNARE-driven fusion (Figure 1)	+	+	+	+	+	↑↑Fusion	↑ Fusion	–	↑↑Fusion
CD (Figure 2)		+				–	↓ Beta (SNAP25)	–	↑Helix (SNAP25) ↑Other (syx)
sdFLIC (Figure 3)			+	+		–	↑*cis* SNARE	–	–
Docking (Figure 4)					+	–	↓↓Docking	–	↓↓Docking
Osmotic-driven fusion (Figure 5)	+				+	↑↑Fusion	↑↑↑Fusion	↓Fusion	NA

+ indicates that a fusion-related interaction was tested with the matching assay for a response from alcohol. – indicates that an assay detected no significant changes for the alcohol and dose listed. Other entries with arrows indicate a significant increase (↑) or decrease (↓) in the listed event.

**FIGURE 6:**
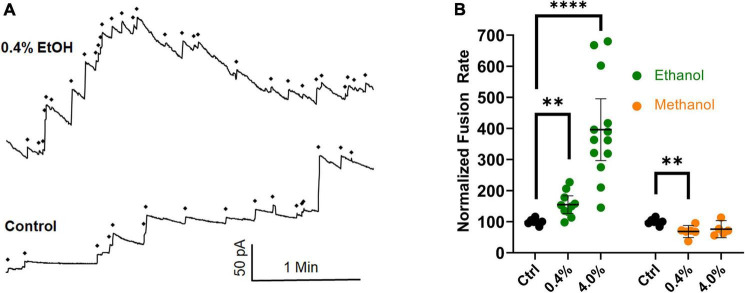
Ethanol (0.4%), increases fusion rates in the protein-free fusion assay over controls. In this assay, an osmotic gradient, not SNARE proteins, is used to drive fusion of liposomes with a planar lipid membrane. **(A)** The control had 17 fusion events and the 0.4% ethanol had 30 fusion events during the 3.5 min segments shown. Each fusion event is marked with a ◆ and is indicated as an abrupt increase in current across the membrane which is voltage clamped at 60 mV. **(B)** Shows the averaged fusion rates of many experiments in response to ethanol (green) and methanol (orange). Error bars are 95% confidence intervals. ^**^p < 0.05 and ^****^p < 0.0001 using two-tailed student’s *t*-test *n* ≥ 5.

New data have been gathered that agree with previously published data (Paxman et al., 2017), in which an osmotic gradient was used as the driving force for vesicle-planar lipid bilayer fusion. The combined data are shown in Figure 6. They show that even in the absence of SNARE proteins, high physiological doses (0.4% or 68 mM) of ethanol increase fusion rates of liposomes to a planar membrane, similar to the results from the SNARE-driven fusion assay. These data are summarized in the last row of Table 1. Taken together, the data strongly support the idea that physiologically relevant doses of ethanol but not methanol increase fusion *via* altering lipid bilayer-bilayer interactions. Ethanol may be acting on lipids by changing lipid-tail splay probability (Stevens et al., 2003; Risselada et al., 2011) or pore formation and expansion (Sharma and Lindau, 2018).

## Discussion

**FIGURE 7:**
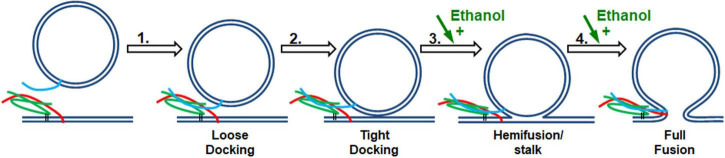
SNARE vesicle fusion process. (1) SNARE assembly leads to Loose Docking. (2) Generation of protein-free membrane patch, overcoming electrostatic repulsion, and dehydration leads to Tight Docking. (3) Lipid tail splaying leads to Hemifusion/stalk formation. (4) Fusion of distal membrane leaflets (pore formation) leads to Full Fusion. Our data suggest that ethanol is acting at transition 3 and/or transition 4. Individual components shown are as in Figure 1.

In considering how ethanol alters neurotransmitter (NT) release from neurons, there are many possible sites where ethanol could act. As discussed in this report, ethanol could act directly on the SNARE proteins and lipid bilayer membranes responsible for the exocytotic release of NT (Figure 1). Additionally, both post-and pre-synaptic mechanisms respond to ethanol. In this study, we used a reductionist approach to look only and specifically at the minimal components of exocytosis involved in NT release. The minimal system contained the three SNARE proteins, proteoliposomes and a supported membrane reconstituted from purified components. With this SNARE-driven fusion assay, we observed that a physiologically high dose of ethanol (0.4% v/v, 68 mM), but not methanol, significantly enhanced the probability of vesicle fusion (Figure 2). This enhancement can only be mediated through the action of alcohol on some components of this minimal system. Table 1 summarizes results from each of the assays reported here. Most relevant are the results for low doses of ethanol, where significant increases in fusion were observed with both the protein-driven and protein-free fusion assays. Figure 7 shows a model that identifies the possible sites of alcohol’s action in exocytosis. Each of these is discussed below.

### Is it SNARE proteins?

If enhancement of fusion is partly or fully due to ethanol acting on proteins, we would expect to see a change in the structure of one or more of the SNARE proteins: syntaxin-1a, SNAP-25A, or synaptobrevin-2, as well as a change in the distance of the acceptor SNARE complex from the membrane due to a more rigid linker of syx between membrane anchor and SNARE motif, or in the probability (or frequency) of vesicle docking. Using CD spectrometry, sdFLIC microscopy, and the TIRF microscopy docking assay, we saw no significant change in any of these results at physiologically relevant concentrations of ethanol (Figures 3–5, respectively). These methods can detect different states of SNAREs and the SNARE proteins would be expected to specifically influence the first two transitions (loose and tight docking) and the last transition (full fusion, Figure 7). While these methods cannot distinguish between loose and tight docking, the results suggest that these protein-dependent transitions are not affected by ethanol. A change was seen with 10% ethanol, but this is likely due to gross changes in the membrane structure or a change in protein structure as seen in the CD spectrometry data (Figure 3). Our results also suggest that ethanol likely has different effects at high doses, which is supported by other experiments in the literature [e.g., (Rowe, 1983)]. Likewise, a high dose of methanol likely affects many of these transitions, since the data show a change in fusion, docking, and secondary structure of SNAP-25 and syx (but not SNARE complex distance from the membrane) at 10% methanol.

### Is it the lipids in membranes?

Since 0.4% ethanol enhances fusion in the SNARE-driven system, but no change could be detected in the SNARE proteins themselves or in the protein-lipid interaction, we conclude that ethanol must be enhancing fusion by its direct action on the lipid components of the membranes, which would most likely affect transitions 3 and 4 in Figure 7. This is consistent with data from the protein-free fusion system (Figure 6). With this assay we observed a significant increase in vesicle-bilayer fusion rates in the total absence of proteins. This increase occurred following the addition of 0.4% ethanol but not methanol (Paxman et al., 2017).

In both the protein-free and SNARE-driven fusion assay, we cannot distinguish between alcohol acting on one or both of the lipid membranes, namely the vesicle or the target membrane. The data suggests that ethanol lowers the activation energy for merging the two bilayers (transitions 3 and 4 in Figure 7). The reduction in activation energy for merging two lipid bilayers can be broken down into two parts; increasing probability of lipid tail splay (that initiates lipid mixing between membranes, transition 3), or by decreasing the energy required for membrane pore formation (transition 4). The enhancement of pore formation by alcohol is consistent with the report by Ly and Longo and supported by results from Wittenberg and colleagues (Ly and Longo, 2004; Wittenberg et al., 2008) that ethanol decreases the energy needed to lyse a vesicle. These combined data are self-supporting that ethanol is acting on lipid membranes to increase exocytosis, regardless of the absence of SNARE proteins.

### Experimental considerations

It is interesting to note that although both fusion assays show a marked increase with 0.4% ethanol, they differ at higher doses. This could be due to the characteristics of each assay and the fact that the SNARE assay measures fusion probability versus fusion rate measured with the protein-free assay. Fusion probability (SNARE assay) declines toward control above 5% ethanol and could be explained by a change in bilayer structure (Simon and McIntosh, 1984) that may alter lipid membrane properties involved in fusing the two membranes. With the protein-free assay, the fusion rate continues to rise substantially as ethanol increases above 0.4% (at 10% the membrane becomes unstable and breaks, ending the experiment; which also suggests a fundamental change in membrane structure). The two assays have basically different dynamic ranges. In other words in the SNARE-driven fusion assay 0.4% ethanol is probably bringing fusion probability from 34% (control) up to its maximum, 60–80% (Kiessling et al., 2013; Kreutzberger et al., 2017a), while in the protein-free assay, fusion probability starts out as low as 2–5% (Woodbury and Hall, 1988a,b; Niles et al., 1989) requiring more ethanol to increase fusion probability (and thus continued increases in fusion rate) to its maximum.

### Application to neurons

As discussed in the introduction, there are many upstream and downstream effects of ethanol on neurons that likely compete with the baseline effects reported here, namely on lipid membranes. Our results are from a simplified system and must underlie any previously reported changes in NT release because they all include cell membranes. Therefore, these results have application to inhibitory (e.g., GABAergic) and excitatory (e.g., Glutamatergic) neurons. Generally, acute alcohol depresses excitatory synapses and potentiates inhibitory synapses (Williams et al., 2018; Roberto et al., 2021). Our data predict that all synapses should be potentiated by ethanol up to a lethal dose (>86 mM). However, synapses that are inhibited by ethanol must be modulated by differences in lipid and/or protein composition not represented in our assays, or other factors not herein considered.

## Conclusion

Our results show that a physiologically relevant dose of ethanol (0.4 vol%, 68 mM) increases the fusion of vesicles to planar membranes in both a protein-free fusion assay and in a SNARE-driven fusion assay. We also show that the conformation of individual SNARE proteins is not significantly changed up to 5% alcohol (as measured in CD spectrometry experiments), and that protein-protein and protein-lipid changes are also not seen until higher alcohol doses (using sdFLIC microscopy). TIRF microscopy-based docking experiments indicate that SNARE engagement persists up to 5% ethanol. This leads us to the conclusion that ethanol mediates an increase in vesicle fusion *via* its effects on the lipid bilayer properties of one or both participating membranes. We would thus predict that if all other known effects of ethanol on proteins in cells were controlled for, addition of ethanol would increase fusion of synaptic vesicles leading to increased neurotransmitter release.

## Data availability statement

The raw data supporting the conclusions of this article will be made available by the authors, without undue reservation.

## Author contributions

RC and DW conceived the idea, wrote the manuscript, and analyzed data for the CD spectrometry and protein-free fusion experiments. VK performed all aspects of the FLIC experiments. AK, KK, and DW performed and analyzed the SNARE fusion experiments. LT oversaw the SNARE fusion, docking, and FLIC experiments. All authors reviewed and edited the manuscript.

## Supplementary material

Supplementary Figure 1 Snapshot of Supplementary Movie 1 from the TIRF microscopy SNARE-driven fusion assay with 0.4% ethanol. Bright dots are single vesicles that are docked at the membrane. Diffuse fluorescent patches are areas where vesicles recently fused and their fluorescent lipids have started to mix with the non-labeled lipids.

Supplementary Figure 2 Representative spectra of the effect of ethanol **(A)** and methanol **(B)** on syntaxin-1a (194–288). The spectra primarily differ in amplitude and deconvolute to similar structural components. Amplitude changes are likely due to precipitation of this shortened version of syntaxin, which is prone to aggregation. The mixed solutions were cloudy and a loss of signal was observable over time and could be recovered by sonicating the sample (data not shown). Because of these properties it is suspected that both alcohols are facilitating precipitation of syx but when sonicated the signal could not be recovered in the presence of >2% alcohol (data not shown).

Supplementary Figure 3 Representative spectra of the effects of ethanol **(A)** and methanol **(B)** on synaptobrevin-2. Alcohol did not affect the secondary structure of syb2 until higher doses.

Supplementary Figure 4 Example sdFLIC images, data fit, and histograms for control **(A)**, and after addition of 0.4% **(B)**, 1% **(C)**, 5% **(D)**, and 10% Ethanol **(E)** to the supported membrane containing Syx*192/SNAP25/Syb(1-96) complex. Column 1: Example images with colored squares marking areas from which intensities were extracted. Scale-bar 20 μm. 50–100 sets of 16 intensities originating from squares of different oxide thickness were extracted from each image. Column 2: Fit of the FLIC theory to one set of 16 oxides. Column 3: Histogram of fit results from one image. For each repeat under each condition, 5 images were acquired and analyzed.

Supplementary Figure 5 Example binding data showing fluorescence intensity increase over time. Square points are individual intensity samples. The smooth curve is the fitted exponential first order kinetic curve used to determine saturation intensity.

Supplementary Movie 1 Time course of an experiment with 0.4% ethanol. Each flash represents a fusion event. Vesicles are not visible until they come within proximity of the TIRF field (i.e. the supported membrane). Vesicles are considered docked when their positions essentially don’t change.
