# Supplementary figures and images for: Drunken lipid membranes, not drunken SNARE proteins, promote fusion in a model of neurotransmitter release
Source: Front Mol Neurosci. 2022 Oct 14;15:1022756. doi: 10.3389/fnmol.2022.1022756 (PMC9614348; doi:10.3389/fnmol.2022.1022756)

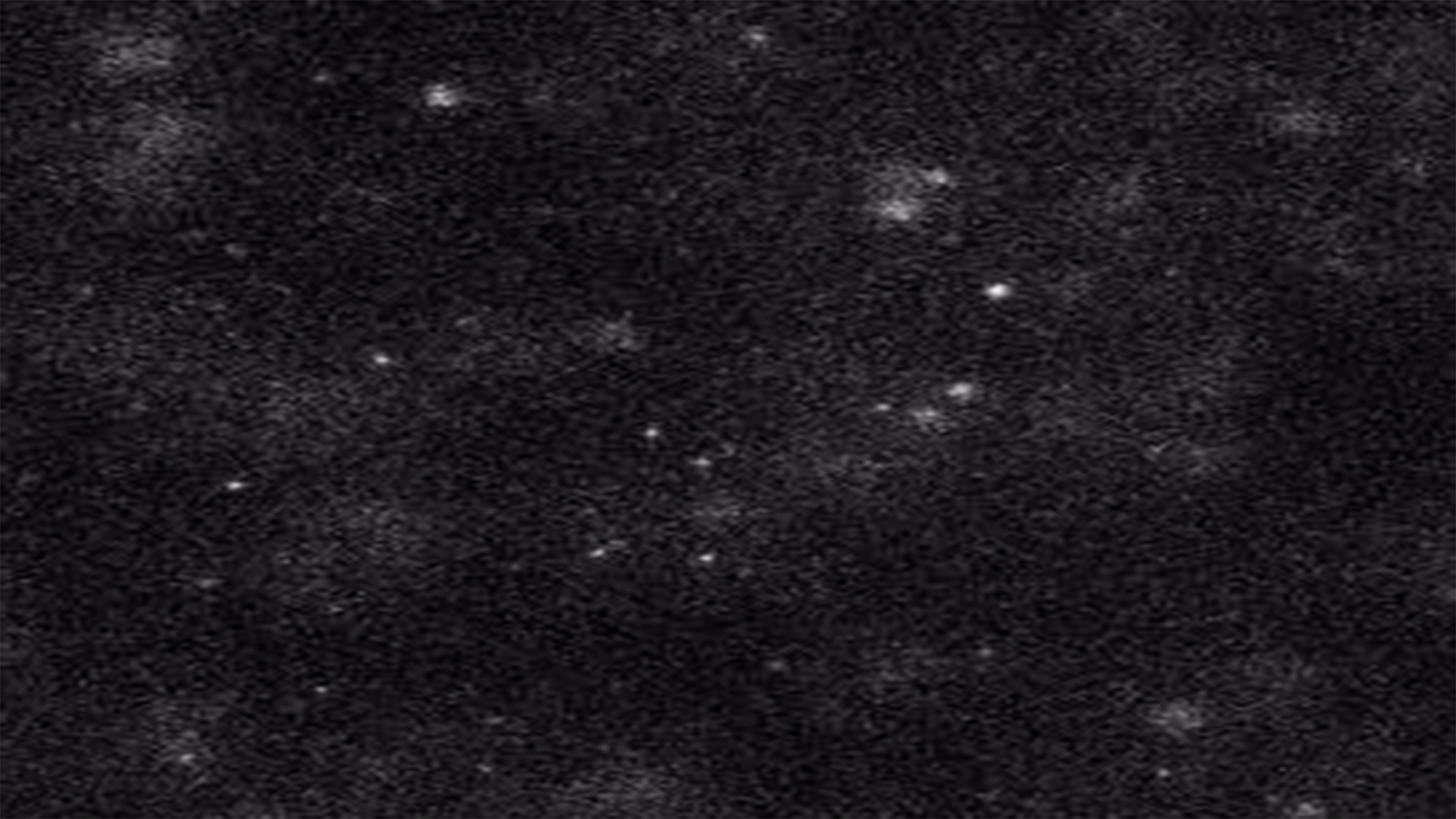

Supplement: Supplementary Figure 1 — Snapshot of Supplementary Movie 1 from the TIRF microscopy SNARE-driven fusion assay with 0.4% ethanol. Bright dots are single vesicles that are docked at the membrane. Diffuse fluorescent patches are areas where vesicles recently fused and their fluorescent lipids have started to mix with the non-labeled lipids. [file Image_1.TIF]

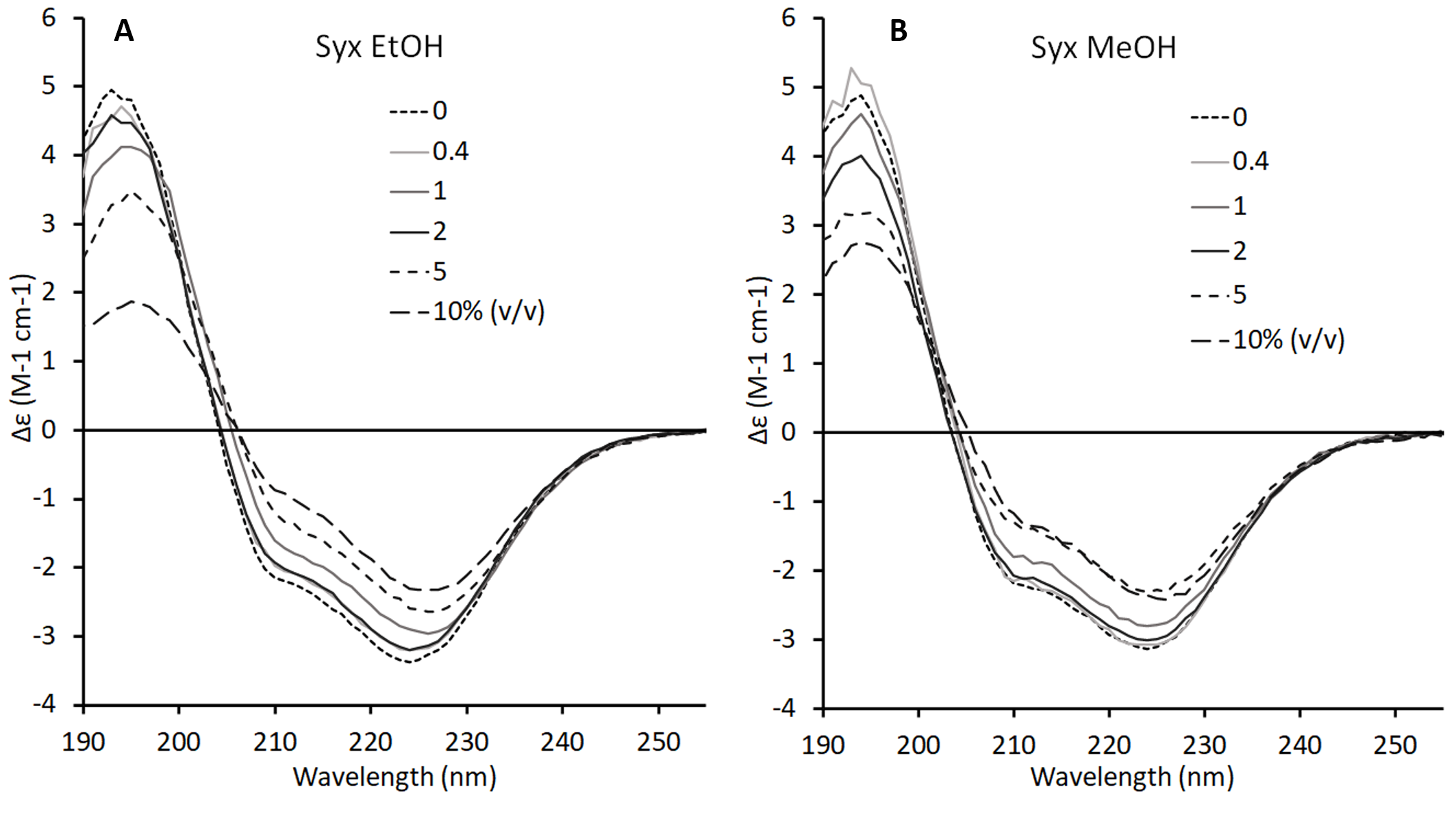

Supplement: Supplementary Figure 2 — Representative spectra of the effect of ethanol (A) and methanol (B) on syntaxin-1a (194–288). The spectra primarily differ in amplitude and deconvolute to similar structural components. Amplitude changes are likely due to precipitation of this shortened version of syntaxin, which is prone to aggregation. The mixed solutions were cloudy and a loss of signal was observable over time and could be recovered by sonicating the sample (data not shown). Because of these properties it is suspected that both alcohols are facilitating precipitation of syx but when sonicated the signal could not be recovered in the presence of >2% alcohol (data not shown). [file Image_2.TIF]

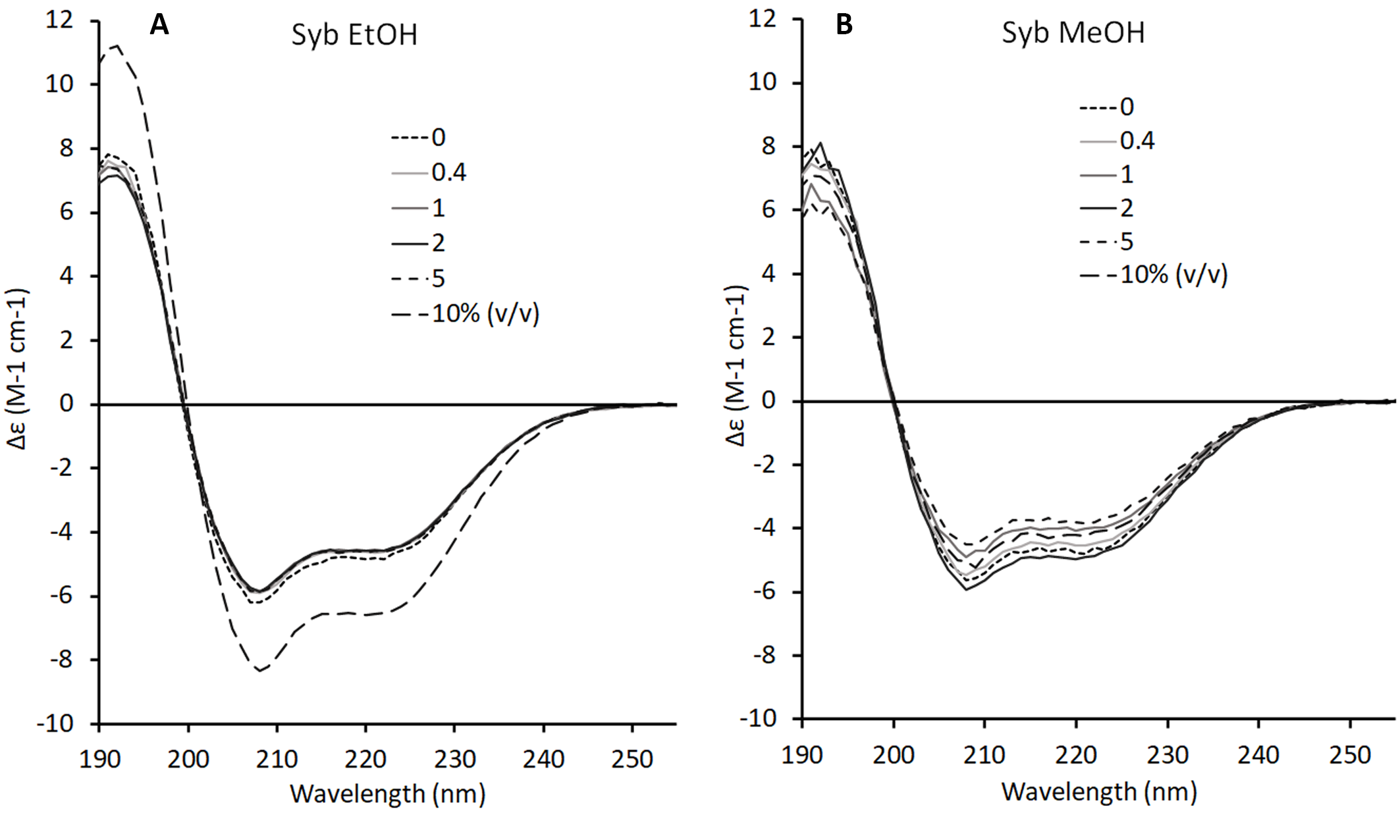

Supplement: Supplementary Figure 3 — Representative spectra of the effects of ethanol (A) and methanol (B) on synaptobrevin-2. Alcohol did not affect the secondary structure of syb2 until higher doses. [file Image_3.TIF]

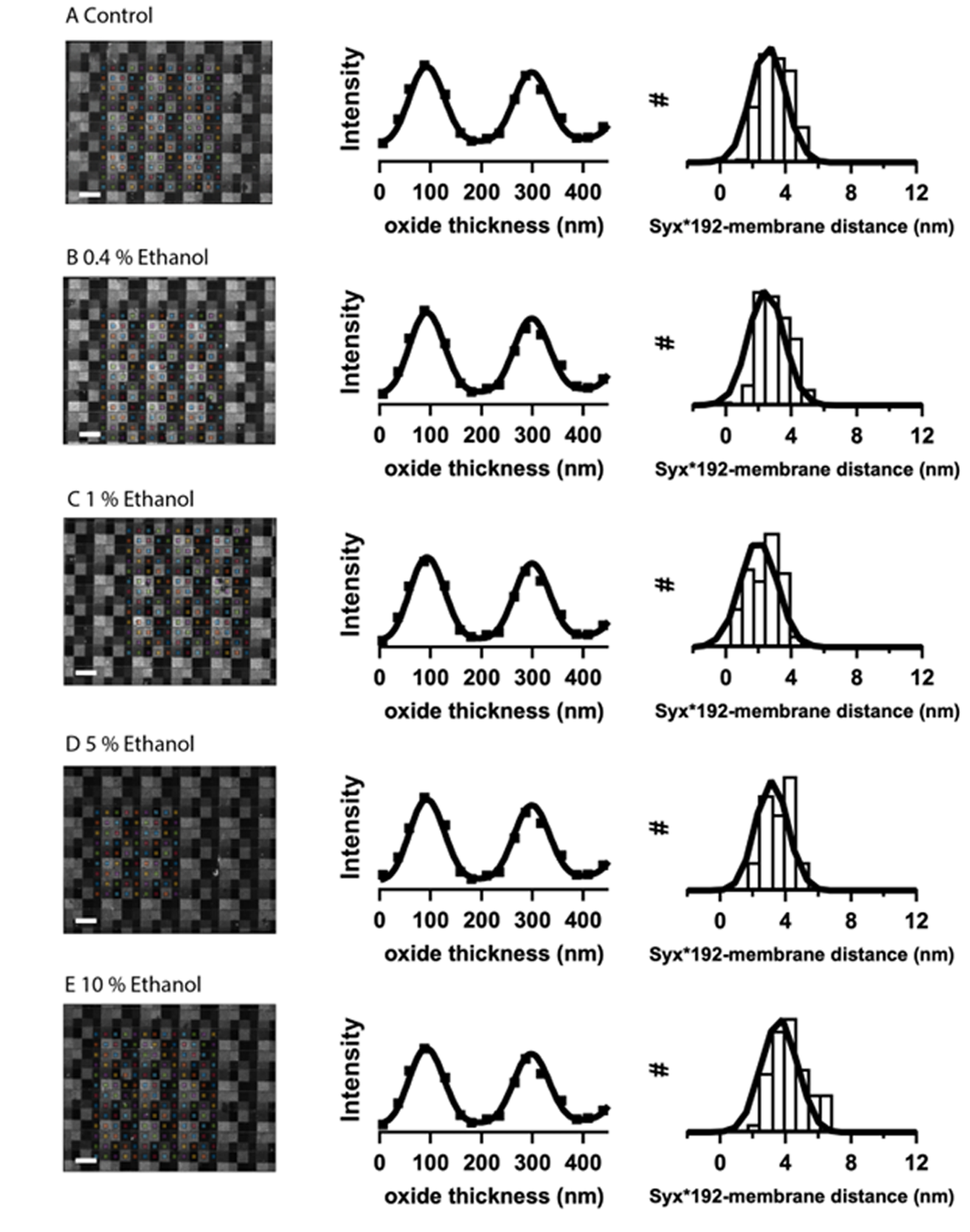

Supplement: Supplementary Figure 4 — Example sdFLIC images, data fit, and histograms for control (A), and after addition of 0.4% (B), 1% (C), 5% (D), and 10% Ethanol (E) to the supported membrane containing Syx*192/SNAP25/Syb(1-96) complex. Column 1: Example images with colored squares marking areas from which intensities were extracted. Scale-bar 20 μm. 50–100 sets of 16 intensities originating from squares of different oxide thickness were extracted from each image. Column 2: Fit of the FLIC theory to one set of 16 oxides. Column 3: Histogram of fit results from one image. For each repeat under each condition, 5 images were acquired and analyzed. [file Image_4.TIF]

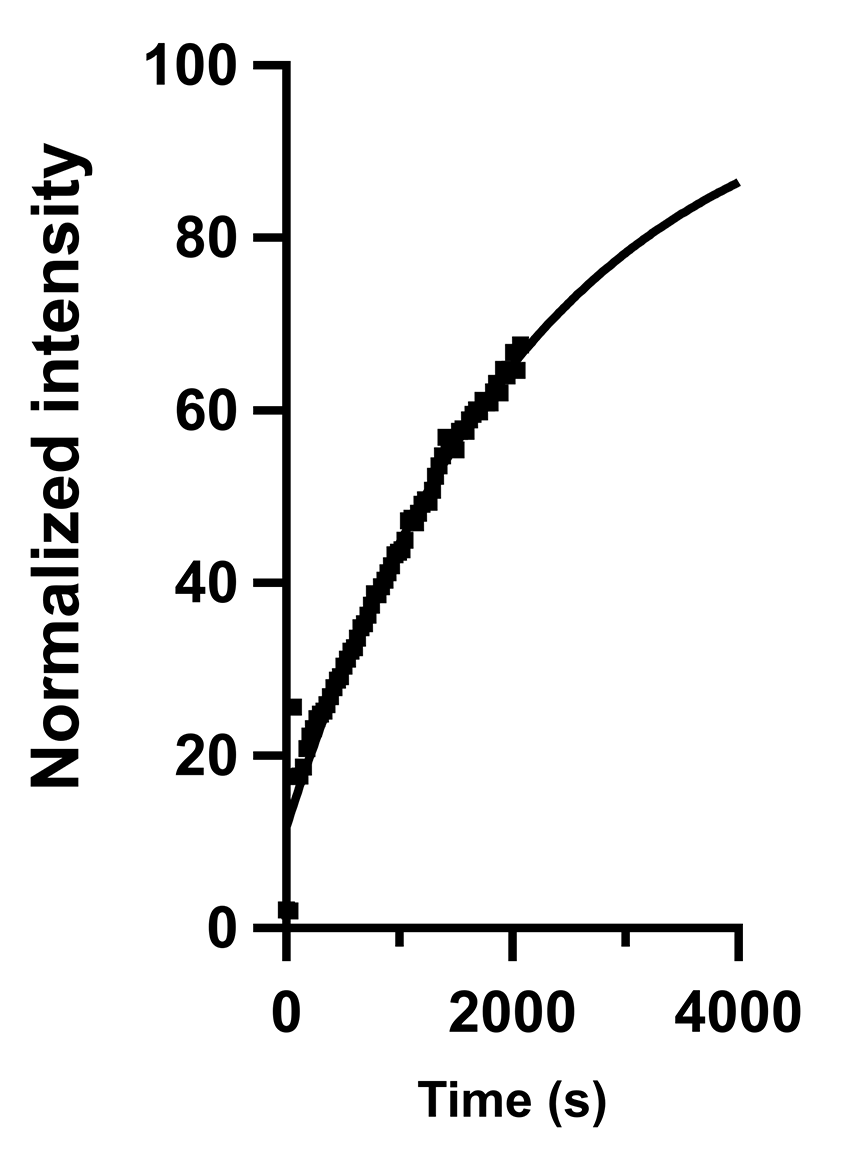

Supplement: Supplementary Figure 5 — Example binding data showing fluorescence intensity increase over time. Square points are individual intensity samples. The smooth curve is the fitted exponential first order kinetic curve used to determine saturation intensity. [file Image_5.TIF]
